# Encapsulated Mixture of Methyl Salicylate and Tributyrin Modulates Intestinal Microbiota and Improves Growth Performance of Weaned Piglets

**DOI:** 10.3390/microorganisms9061342

**Published:** 2021-06-21

**Authors:** Yusen Wei, Jiangdi Mao, Jingliang Liu, Yu Zhang, Zhaoxi Deng, Jiaqi Lv, Maolong He, Jianxin Liu, Haifeng Wang

**Affiliations:** 1 The Key Laboratory of Molecular Animal Nutrition, Ministry of Education, College of Animal Science, Zhejiang University, Hangzhou 310058, China; yusenwei@zju.edu.cn (Y.W.); jiangdimao@126.com (J.M.); jingliangliu@zju.edu.cn (J.L.); zhangyuu@zju.edu.cn (Y.Z.); zhaoxideng@zju.edu.cn (Z.D.); liujx@zju.edu.cn (J.L.); 2 Lucta SA., UAB Research Park, Edifici Eureka, 08193 Bellaterra, Spain; louis.lv@lucta.com (J.L.); maolong.he@lucta.com (M.H.)

**Keywords:** tributyrin, oregano essential oil, methyl salicylate, growth performance, microbiota, metabolomics, piglets

## Abstract

Tributyrin and essential oils have been used as alternatives to antimicrobials to improve gut health and growth performance in piglets. This study was to evaluate the effects of a dietary supplement with two encapsulated products containing different combinations of tributyrin with oregano or with methyl salicylate on growth performance, serum biochemical parameters related to the physiological status, intestinal microbiota and metabolites of piglets. A total of 108 weaned crossbred piglets (Yorkshire × Landrace, 21 ± 1 d, 8.21 ± 0.04 kg) were randomly divided into three groups. Piglets were fed with one of the following diets for 5 weeks: a basal diet as the control (CON); the control diet supplemented with an encapsulated mixture containing 30% of methyl salicylate and tributyrin at a dosage of 3 kg/t (CMT); and the control diet supplemented with an encapsulated mixture containing 30% of oregano oil and tributyrin at a dosage of 3 kg/t (COT). At the end of the feeding trial, six piglets from each group were slaughtered to collect blood and gut samples for physiological status and gut microbiological analysis. The study found that the CMT group was larger in feed intake (FI) (*p* < 0.05), average daily gain (ADG) (*p* = 0.09), total protein (TP), albumin (ALB), glutathione peroxidase (GSH-PX) (*p* < 0.05), blood total antioxidant capacity (T-AOC) (*p* < 0.05), and crypt depth in the ileum (*p* < 0.05) compared with the CON group. The genus abundance of *Tissierella* and *Campylobacter* in the CMT group was significantly decreased compared with the CON group. The CMT group also resulted in significantly higher activity in amino acid metabolism and arginine biosynthesis compared with the CON group. The COT group was larger in T-AOC, and the genus abundance of *Streptophyta* and *Chlamydia* was significantly increased in the ileum compared with the CON group. Data analysis found a significantly high correlation between the genus abundance of *Chlamydia* and that of *Campylobacter* in the ileum. The genus abundance of *Campylobacter* was also positively correlated with the sorbitol level. In general, the results indicated that the supplementation of both encapsulated mixtures in diet of weaned piglets could improve the animal blood antioxidant capacity. Additionally, the encapsulated mixture of methyl salicylate plus tributyrin improved the growth performance and resulted in certain corresponding changes in nutrient metabolism and in the genus abundance of ileum microbial community.

## 1. Introduction

There were growing evidence that the stress response caused by weaning would destroy the anti-oxidative balance [1], caused intestinal inflammation and damaged the intestinal barrier, furthermore worsened the absorptive capacity of the small intestine [2], which would decrease growth performance and eventually cause economic losses [3].

The compatibility of organic acids and plant essential oils to mitigate the negative effects of weaning stress at a lower cost had attracted much attention [4]. Butyric acid as a short-chain fatty acid had a certain therapeutic effect on intestinal diseases by improving intestinal morphology and reducing the rate of apoptosis [5]. Tributyrin, as a source of butyrate, that one molecule releases three molecules of butyrate directly in the small intestine, could be used as an effective feed additive for weaned piglets to improve performance and intestinal health of piglets [6]. Compared with other butyrates, tributyrin has acceptable organoleptic characteristics and provides a higher and slowly released source of butyric acid in the small intestine [7].

Oregano oil was derived from the natural plant oregano (*Origanumvuelgare L.*) [8]. The main components of the oregano oil extracted from leaves and flowers were phenolic compounds, in which carvacrol and thymol had great antibacterial and antioxidant properties [9]. Dietary inclusion of oregano oil has many benefits for the weaned piglets, including strengthening immune response, improving antioxidant capacity and relieving stress [10]. Methyl salicylate was also a kind of essential oil extracted from holly (*I. purpurea Hassk.*), which has been used for its bactericidal and anti-inflammatory effects [11]. There are few reports on the supplementation of methyl salicylate in animal diets. Additionally, considerable attention has been paid to the potential use of organic acids and plant essential oils to look for benefits in the animal.

Based on our previous study, tributyrin at dosage of 1.4 g per kilogram combined with oregano essential oil (OT) or methyl salicylate (MT) respectively 0.6 g per kilogram in powered form may have beneficial effects at the intestinal level of piglets, including the morphological structure and microbiota communities [12]. However, both treatments did not show significant differences in performance parameters. Here, we introduced the encapsulated mixture of essential oil and tributyrin to avoid the lipophilicity and volatility of plant essential oil. This study was aimed to investigate the role of dietary inclusion of encapsulated mixture of tributyrin and essential oil or methyl salicylate in a granular form to modulate the gut microbiota, metabolomics and intestinal morphology, and improving serum antioxidant capacity and growth performance of weaned piglets.

## 2. Materials and Methods

### 2.1. Animals and Experimental Diets

A total of 108 weaned piglets (Yorkshire × Landrace, 21 ± 1 d, body weight 8.21 ± 0.04 kg), were allowed to three groups according to sex and weight, with 6 pens for each group and 6 piglets for each pen. The piglets were fed with a base diet as the control (CON), an antibiotic-free basal diet composed based on NRC weaned piglet nutritional standards, or the control supplemented with an encapsulated mixture containing 30% of methyl salicylate and tributyrin at a dosage of 3 kg/t (CMT); and the control diet supplemented with an encapsulated mixture containing 30% oregano oil and tributyrin at a dosage of 3 kg/t (COT). Encapsulation was made mainly with calcium palmitate and the sample products were provided by Lucta (Guangzhou) Flavours Co., Ltd. (Guangdong, China). All animal procedures were performed in full accordance with the Regulation for the Use of Experimental Animals in Zhejiang Province, China. This work was specifically approved by the Animal Care and Use Committee of Zhejiang University (ethics code permit no. ZJU20170529). The basic diet composition and nutritional level are shown in Table 1. The experiment lasted for 35 days. The body weight was individually measured on day 0 and day 35. Feed consumption was recorded every 3 days until the end of the experimental period. At day 35, six piglets were selected from each group and euthanized by intra-abdominal injection of 200 mg/kg pentobarbital sodium to collect biological samples.

### 2.2. Collection of Blood and Intestinal Samples

Blood samples were collected using a coagulation tube, centrifuged at 3000× *g* and 4 °C for 15 min. Serum was collected and stored at −20 °C. Ileum and colonic tissues and contents were collected, and immediately frozen in liquid nitrogen and stored at −80 °C. Ileum and colon tissue were also fixed with phosphate-buffered paraformaldehyde (4%, pH 7.6) for further histological measurements. The pH value of the ileum and colon contents was determined immediately.

### 2.3. Biochemical Analysis and Anti-Oxidatant Capacity Related Parameters of Serum

Concentration of serum albumin (ALB), globulin (GLB), albumin/globulin(A/G), glutamic-pyruvic transaminase (ALT), glutamic-oxalacetic transaminase (AST), glucose (GLU), lactate dehydrogenase (LDH), total cholesterol (TC), triglycerides (TG), total protein (TP), uric acid (UA) and blood urea nitrogen (BUN) were determined by automatic blood cell analyzer (XN-2000-A1, SYSMEX, Kobe, Japan). Anti-oxidant capacity related blood parameters included methylene dioxyamphetamine (MDA), superoxide dismutase (SOD), glutathione peroxidase (GSH-PX) and blood total antioxidant capacity (T-AOC), which were measured using commercial kits (Nanjing Jiancheng Bioengineering Institute, Nanjing, China) according to the manufacturer’s instructions).

### 2.4. Morphological Analysis of Ileum

The ileum tissue of the piglet was immersed in 10% neutral formalin and covered with wax. The waxed tissue block was manually cut into 3 μm-thick sections, and subjected to deparaffinization and dehydration. Then these sections were treated with different concentrations of alcohol (100%, 95% and 75%) for 15 min, and stained with H&E [13]. An optical microscope system (Olympus Corporation, Tokyo, Japan) was used to obtain a micrograph at a combined magnification of 100× using ImageJ software (National Institute of Health, Bethesda, MD, USA). Villus height and crypt depth were measured according to a described method [3]. The ratio of villus height to crypt depth (VH/CD) was calculated.

### 2.5. Analysis of Short-Chain Fatty Acid in Colonic Content

A slightly modified method was used to detect short-chain fatty acids (SCFAs). Colonic contents (0.4 g) were mixed and vortexed with 1.5 mL of phosphate-buffered saline (PBS). The sample was centrifuged at 15,000× *g* for 15 min at 4 °C. The supernatant was added with 25% metaphosphoric acid at a ratio of 9:1 (*v*/*v*), and then the supernatant was passed through a 0.22 μm filter membrane to prepare a colon sample for short-chain fatty acid (SCFA) analysis. A gas chromatograph (model: GC-2010; Shimadzu Corp, Kyoto, Japan) equipped with a chromatographic column (HP-INNOWAX (19091N-133), 30 m × 0.25 mm × 0.25 μm) was used to determine the VFA concentration in the sample [14].

### 2.6. 16S rRNA Sequencing Analysis

Microbial DNA was extracted from ileum contents using the QIAamp Fast DNA Stool Minikit (Qiagen, Hilden, Germany) according to manufacturer’s protocols. The V3-V4 region of the bacteria 16S rRNA genes were amplified by PCR (95 °C for 3 min, followed by 30 cycles at 98 °C for 20 s, 58 °C for 15 s, and 72 °C for 20 s and a final extension at 72 °C for 5 min) using primers 341F 5’-CCTACGGGRSGCAGCAG)-3’ and 806R 5’-GGACTACVVGGGTATCTAATC-3′. PCR reactions were performed in 30 μL mixture containing 15 μL of 2 × KAPA Library Amplification ReadyMix, 1 μL of each primer (10 μM), 50 ng of template DNA and ddH_2_O. Amplicons were extracted from 2% agarose gels and purified using the AxyPrep DNA Gel Extraction Kit (Axygen Biosciences, Union City, CA, USA) according to the manufacturer’s instructions and quantified using Qubit^®^ 2.0 (Invitrogen, Carlsbad, CA, USA). After preparation of library, these tags were sequenced on MiSeq/HiSeq platform (Illumina, Inc., San Diego, CA, USA) for paired-end reads of 500/250 bp. DNA extraction, library construction and sequencing were conducted at Realbio Genomics Institute (Shanghai, China).

Tags, trimmed of barcodes and primers, were further checked on their rest lengths and average base quality. 16S tags were restricted between 220 bp and 500 bp such that the average Phred score of bases was no worse than 20 (Q20) and no more than 3 ambiguous N. The copy number of tags was enumerated and redundancy of repeated tags was removed. Only the tags with a frequency of more than 1, which tend to be more reliable, were clustered into OTUs, each of which had a representative tag. Operational taxonomic units (OTUs) were clustered with 97% similarity using UPARSE (http://drive5.com/uparse/; accessed on 11 November 2020) [15] and chimeric sequences were identified and removed using Userach (version 7.0). Each representative tags was assigned to a taxa by RDP Classifer (http://rdp.cme.msu.edu/; accessed on 11 November 2020) [16,17] against the RDP database (http://rdp.cme.msu.edu/; accessed on 11 November 2020) using a confidence threshold of 0.8. OTU profiling table and alpha/beta diversity analyses were also achieved by python scripts of QIIME (V1.9.1). Anosim was analyzed for the microbial community structure, and Unifrac algorithm was used to calculate the distance between the two samples. Heatmap was created using R (V3.5.1) “g plots” package. Spearman correlation heat map was drawn by the R (V3.5.1) “corrplot” package. PICRUSt (phylogenetic investigation of communities by reconstruction of unobserved states) is based on 16S rRNA and reference sequence database to predict the functional composition of metagenomics.

### 2.7. GC-TOF-MS and Metabolomics Data Processing

After extracting metabolites [18], all ileum contents were analyzed by GC-TOF-MS analysis performed using an Agilent 7890 gas chromatograph coupled with a time-of-flight mass spectrometer (J&W Scientific, Folsom, CA, USA). The system was equipped with a DB-5MS capillary column. One microliter aliquot of sample was injected in splitless mode. Helium was used as the carrier gas, the front inlet purge flow was 3mL min^−1^, and the gas flow rate through the column was 1 mL min^−1^. The initial temperature was kept at 50 °C for 1min, then raised to 310 °C at a rate of 10 °C min^−1^, then kept for 8 min at 310 °C. The injection, transfer line, and ion source temperatures were 280 °C, 280 °C and 250 °C, respectively. The energy was −70 eV in electron impact mode. The mass spectrometry data were acquired in full-scan mode with the m/z range of 50–500 at a rate of 12.5 spectra per second after a solvent delay of 6.35 min.

Raw data analysis, including peak extraction, baseline adjustment, deconvolution, alignment and integration, was finished with Chroma TOF (V 4.3x, LECO) software and LECO-Fiehn Rtx5 database was used for metabolite identification by matching the mass spectrum and retention index [18]. Finally, the peaks detected in less than half of QC samples or RSD > 30% in QC samples were removed. Then, the missing values were filled up by half of the minimum value. Internal standard normalization method was employed in this data analysis. Data were scaled and logarithmically transformed to minimize the impact of both noise and high variance of the variables. The final dataset containing the information of peak number, sample name and normalized peak area was imported to the MetaboAnalyst 4.0 (http://www.metaboanalyst.ca/; accessed on 10 January 2021) for multivariate analysis. Orthogonal partial least squares discrimination analysis (OPLS-DA), and *t*-test were performed between the two groups, with the FDR adjusted *p* value < 0.05 and the VIP > 1.5 being considered as significantly different metabolites. In addition, commercial databases including KEGG (http://www.genome.jp/kegg/; accessed on 10 January 2021) were used for pathway enrichment analysis.

### 2.8. Statistical Analysis

Sample sizes were chosen empirically based on previous experiments. The piglet was used as the experimental unit for blood, intestinal and microbial parameters, and data from the pen for performance parameters. Data was analyzed by using SPSS 20 (SPSS Inc., Chicago, IL, USA). When the data satisfies the normal distribution and homogeneity of variance, one-way of variance (ANOVA) was conducted and means were further compared by using Tukey’s multiple comparison tests. The non-parametric Kruskal–Wallis test with Dunn’s multiple comparisons test was used for a date that was not normally distributed. Results are reported as means ± standard error of the mean (SEM). *p* < 0.05 was considered as significant indicated by “*” and 0.05 < *p* < 0.1 was considered as a tendency.

## 3. Results

### 3.1. Growth Performance

**Figure 1:**
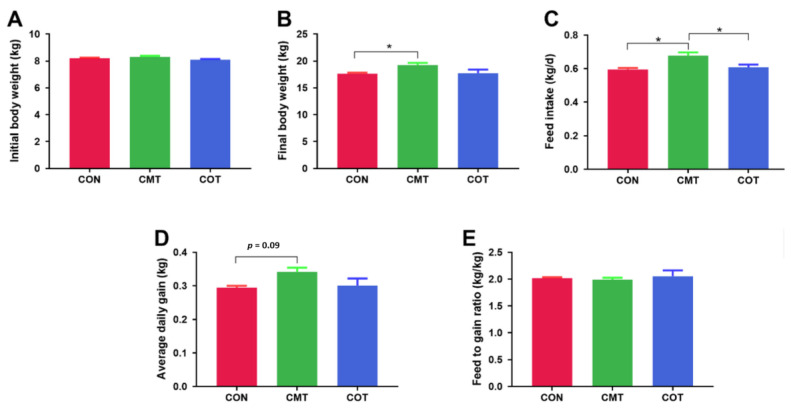
Growth performance of piglets in different treatments. (**A**) Initial body weight. (**B**) Final body weight. (**C**) Feed intake. (**D**) Average daily gain. (**E**) Feed to gain ratio. Star (*) indicates that there are significantly different (at the 5% threshold of the ANOVA) between groups.

The average body weight of piglets increased from 8.21, 8.10 and 8.31 kg at initial for the CON, COT and CMT groups (Figure 1A), respectively, to 17.64, 17.74 and 19.24 kg in the end (Figure 1B). The CMT group was larger in the FI compared with the CON group (*p* < 0.05) and the COT group (*p* < 0.05), which increased by 13.9% and 11.6% respectively (Figure 1C). These correspondingly tended to have larger ADG compared with the CON group (*p* = 0.09), which increased by 15.9% (Figure 1D). Compared with the CON group, the COT group only slightly increased the FI and the ADG by 2.1% which were not significant. The difference on feed to gain ratio among the groups was not significant, although the CMT group slightly reduced it by 1.5% whereas the COT group increased it by 1.7% compared with the CON group (Figure 1E).

### 3.2. Serum Biochemical Indices and Anti-Oxidative Capacity Parameters

**Figure 2:**
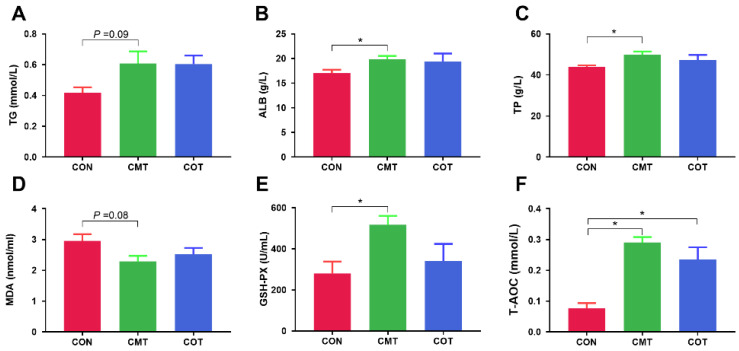
Effects of COT and CMT treatments on serum indices of piglets. (**A**) TG. (**B**) ALB. (**C**) TP. (**D**) MDA. (**E**) GSH-PX. (**F**) T-AOC. Star (*) indicates that there are significantly different (at the 5% threshold of the ANOVA) between groups.

There were no significant differences in A/G, LDH, TC, GLU, ALT, AST, BUN, UA among the three groups (Figure S1A–H). The content of TG tended to increase in the CMT group compared with the CON (*p* = 0.09, Figure 2A). ALB and TP content were significantly higher in the CMT group than the CON group (*p* < 0.05, Figure 2B,C). MDA level had a decreased tendency in the CMT group compared with the CON group (*p* = 0.08, Figure 2D). There was no significant difference in SOD among different treatments (Figure S1I). Compared with the CON group, the CMT group had significantly higher GSH-PX (*p* < 0.05, Figure 2E) and T-AOC (*p* < 0.05, Figure 2F), and the COT group also had significant higher T-AOC level (*p* < 0.05, Figure 2F).

### 3.3. Ileum Morphological Analysis

**Figure 3:**
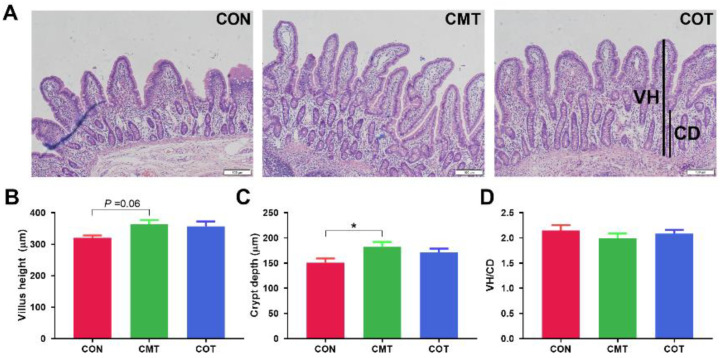
Histological and morphometrical analyses of ileum. (**A**) Hematoxylin and eosin staining of ileum tissue. (**B**) Villus height. (**C**) Crypt depth. (**D**) The ratio of VH/CD. 100×magnification; Scale bars, 100 µm. Star (*) indicates that there are significantly different (at the 5% threshold of the ANOVA) between groups.

The epithelial structure of the ileum in piglets from each group was normal, and the intestinal mucosa was intact without significant pathological changes (Figure 3A). The CMT group had an increased trend in villus length compared with the CON group (*p* = 0.06, Figure 3B). The crypt depth was significantly higher in the CMT group than in the CON group (*p* < 0.05, Figure 3C). There was no significant change in the ratio of VH/CD among the different groups (Figure 3D).

### 3.4. Intestinal Content pH and Colonic SCFAs Profile

**Figure 4:**
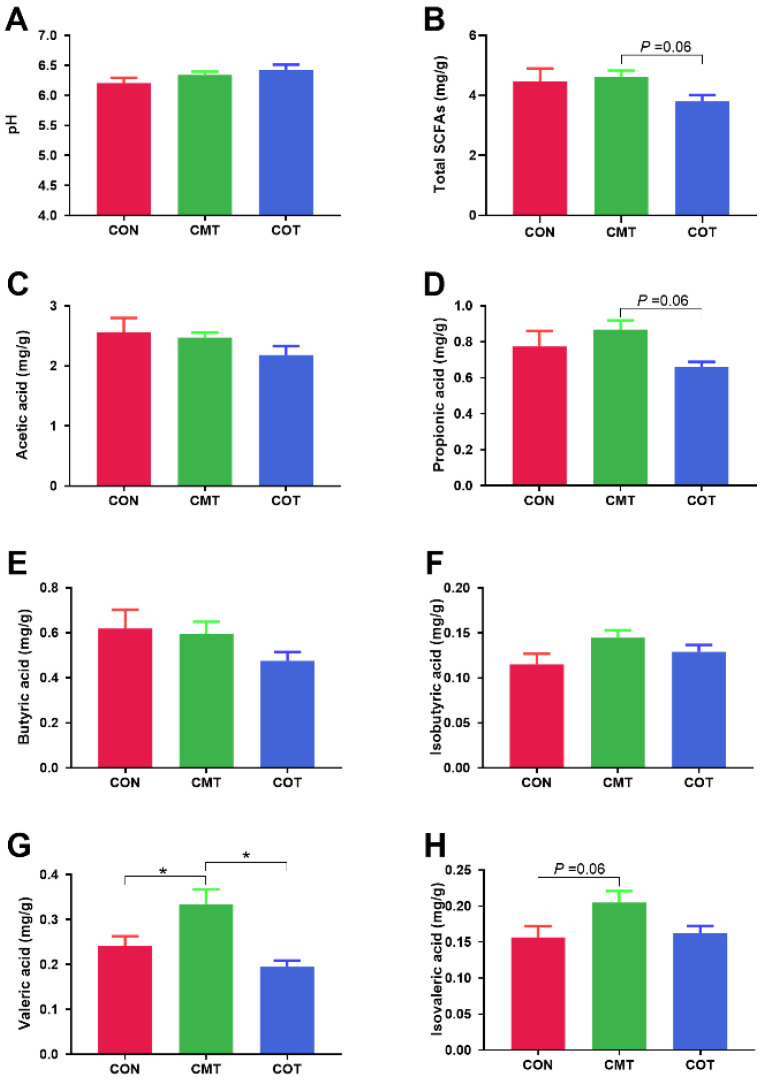
Effects of COT and CMT treatments on SCFAs in colon. (**A**) The value of pH. (**B**) Total SCFAs. (**C**) Acetic acid. (**D**) Propionic acid. (**E**) Butyric acid. (**F**) Isobutyric acid. (**G**) Valeric acid. (**H**) Isovaleric acid. Star (*) indicates that there are significantly different (at the 5% threshold of the ANOVA) between groups.

There was no significant difference in the pH value of the colon of the piglets among the three groups (Figure 4A). The CMT group had an increased trend in the content of total SCFAs (*p* = 0.06, Figure 4B) and propionic acid compared with the COT group (*p* = 0.06, Figure 4D). The CMT group had a significantly higher valeric acid content than the CON group (*p* < 0.05, Figure 4G) and the COT group (*p* < 0.05, Figure 4G). The CMT group also had an increased trend in the isovaleric acid content compared with the CON group (*p* = 0.06, Figure 4H).

### 3.5. Compositional Profiles of the Intestinal Microbiota

**Figure 5:**
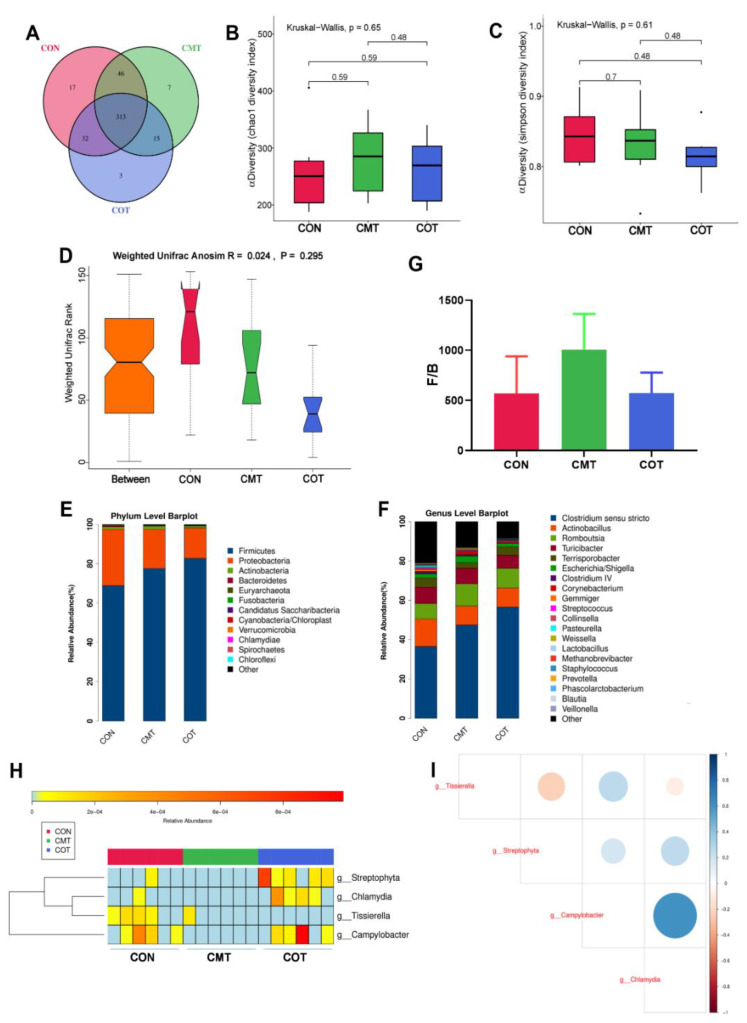
The high-throughput sequencing of 16S rRNA in ileum contents of piglets. (**A**) Venn analysis of bacterial OTUs composition among the three groups. (**B**) Chao1 index. (**C**) Simpson index. (**D**) ANOSIM, analyses of similarities among the three groups of samples. (**E**) Proportional taxonomic assignments at the phylum level from the three groups. (**F**) Proportional taxonomic assignments at the genus level from three groups. (**G**) The ratio of F/B among the three groups. (**H**) Heatmap cluster analysis of differential bacteria at genus level which has a contribution to group differences. (**I**) Spearman correlation analysis of the differential bacteria.

Venn diagram showed that there were 359 and 345 common bacterial groups between the CON group and the CMT group, and between the CON group and the COT group, respectively. There were 17, 3 and 7 unique taxa in the CON, COT, CMT groups, respectively (Figure 5A) determined by Wilcoxon signed-rank test. No significant difference was found in alpha diversity such as Chao1 index and Simpson index among the three groups (Figure 5B,C). The visualization of beta diversity showed that the experimental treatment is meaningful (R > 0, Figure 5D). The relative abundance of bacterial taxa at the phylum (Figure 5E) and genus level (Figure 5F) in piglets from different groups were shown. The phyla *Firmicutes* showed a tendency to increase in abundance in the experimental groups compared with the CON group (Figure 5E). The genus of *Clostridium sensu stricto* also showed a tendency to increase in abundance in the experimental groups compared with the CON group (Figure 5F). The ratio of *Firmicutes/Bacteroidetes* (F/B) tended to increase in the CMT group compared with the CON group (Figure 5G). The heatmap summarized the differences of three groups of bacteria at the genus level namely *Streptophyta*, *Chlamydia*, *Tissierella* and *Campylobacter* (Figure 5H). Spearman correlation test showed that there was a positive correlation between *Campylobacter* and *Chlamydia* (Figure 5I).

A total of 190 Kos (Figure S2A) and 50 MetaCyc pathways (Figure S2B) were identified with significant differences among the three groups. Compared with the CON group, the expressions of KO0059, KO0626, KO1692, KO0249 and other genes related to fatty acid metabolism in the CMT and the COT groups were up-regulated (Figure S2C). The expression of quorum sensing and arginine biosynthesis-related genes such as KO2035, KO2034, KO2032, KO2033 and KO5597 were up-regulated in the CMT group compared with the CON group (Figure S2C). We found that the expression of toxin-related genes such as K18839, K10953 and K10938 were up-regulated in the COT group compared with the CMT group (Figure S2C).

### 3.6. Overview of the Intestinal Metabolome

**Figure 6:**
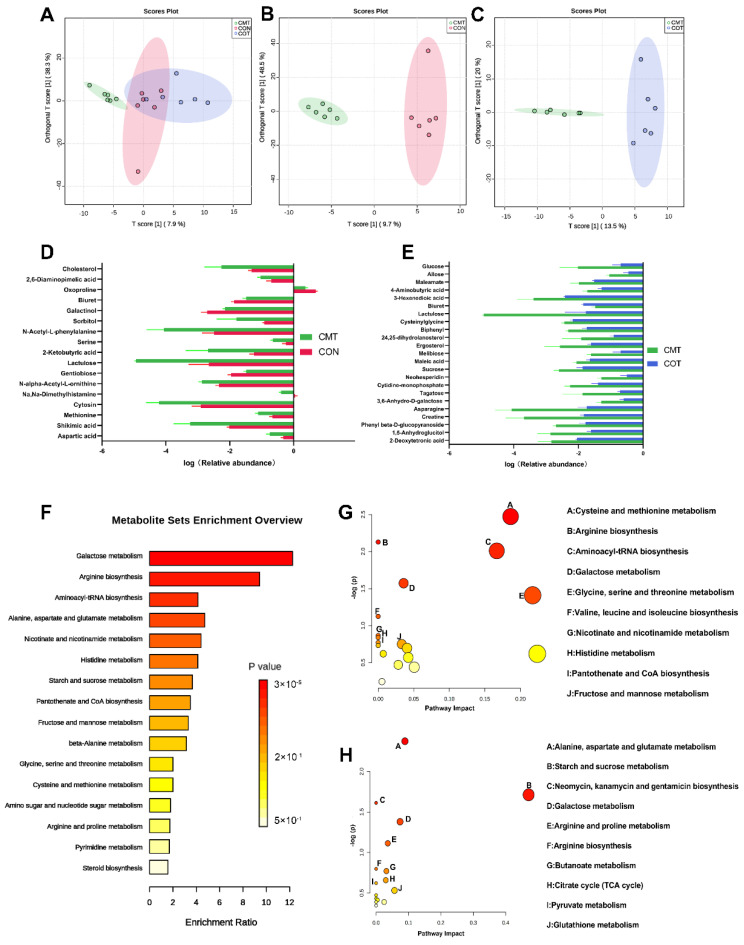
Non-targeted metabonomics derived from the GC/MS metabolite profiles of ileum contents. (**A**) OPLS-DA score map of separate comparisons among the three groups. (**B**) OPLS-DA score map of separate comparisons between the CMT group and the CON group. (**C**) OPLS-DA score map of separate comparisons between the CMT group and the COT group. (**D**) Significantly differential metabolites between the CON group and the CMT group. (**E**) Significantly differential metabolites between the COT group and the CMT group. (**F**) Differential metabolites were classified by KEGG pathway enrichment and significance analysis. (**G**) Pathway enrichment analysis performed using the significantly different intestinal metabolites between the CON group and the CMT group. (**H**) Pathway enrichment analysis performed using the significantly different intestinal metabolites between the COT group and the CMT group.

In this study, 686 peaks were detected and 482 metabolites were left after relative standard deviation de-noising. Supervised orthogonal projections to latent structures-discriminate analysis (OPLS-DA) were applied (Figure 6A–C) to visualize group separation and find significantly changed metabolites. The OPLS-DA model among different groups were as follows: R_2_X = 0.653, R_2_Y = 0.824, and Q_2_ = 0.279 (Figure 6A). The OPLS-DA model between the CMT group and the CON group were as follows: R_2_X = 0.67, R_2_Y = 0.973 and Q_2_ = 0.632 (Figure 6B). The OPLS-DA model between the CMT group and the COT group were as follows: R_2_X = 0.336, R_2_Y = 0.918, and Q_2_ = 0.568 (Figure 6C). The Q_2_ values all exceeded 0.4, indicating that these models were more reliable and that consistent modeling and predictability were achieved. However, the OPLS-DA model between the COT group and the CON group were as follows: R_2_X = 0.626, R_2_Y = 0.646, and Q_2_ = −0.0417 (Figure S3A). Subsequently, we detected 25 differential metabolites among the three groups. There were 17, 23 and 11 differential metabolites in the CON, CMT and COT groups, respectively (Figure 6D,E, Figure S3B).

Exact mass data (m/z) from the KEGG and HMDB databases were used to annotate the differential metabolites for pathway enrichment analysis (Figure 6F). Differential metabolic pathways between each two group were shown (Figure 6G,H). The significant differences in the expression of molecules were associated with galactose metabolism, arginine biosynthesis, aminoacyl-tRNA biosynthesis, alanine, aspartate and glutamate metabolism during the trial period. The level of methionine, serine, 2-ketobutyric acid related to cysterine and methionmine metabolism in the CMT group were significantly lower than in the CON group. Regarding arginine biosynthesis, the concentrations of aspartic acid and N-alpha-Acety-L-ornithnine in the CMT group were significantly lower than that in the CON group. Concerning aminoacyl-tRNA biosynthesis, aspartic acid in the CMT group was also less than that in the CON group. Compared with the CMT group, the COT group had a significantly higher concentration of glucose related to starch and sucrose metabolism and neomycin, kanamycin and gentamicin biosynthesis, and also had a significantly higher level of aspartic acid, maleic acid and 4-aminobutyric related to alanine, aspartate and glutamate metabolism.

### 3.7. Correlation of Characteristic Gut Microbiota and Metabolites

**Figure 7:**
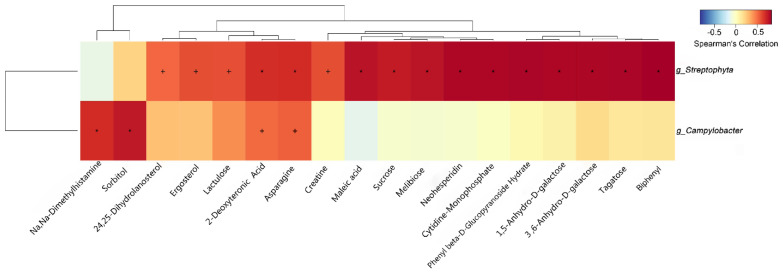
Heatmap of Spearman correlation analysis between the altered microbiota and metabolites.

The correlation between differential gut bacteria and metabolites among the three groups was expounded by Spearman correlation analysis (Figure 7). The genus *Streptophyta* was strongly positively correlated with 2-deoxytetronic acid, aspartic acid, melibiose, maleic acid, sucrose, neohesperidin, phenyl β-D-glucopyranoside hydrate, cytidine monophosphate, 1,5-anhydroglucitol, 3,6-anhydro-D-galactose, tagatose and biphenyl (*p* < 0.05), and highly positively correlated with 24,25-dihydrolanosterol, ergosterol, lactulose and creatine (*p* < 0.05). The genus *Campylobacter* was strongly positively correlated with Na, Na-dimethylhistamine and sorbitol (*p* < 0.05), and highly positively correlated with 2-deoxytetronic acid and aspartic acid (*p* < 0.05).

## 4. Discussion

Plant essential oil which had many beneficial effects on animal health has been excavated [19]. Tributyrin was a new way to provide butyric acid, which was more efficient in intestinal utilization than butyric acid [20]. Using the application of coating technology could solve the disadvantage that essential oil could not reach the intestinal tract effectively [21]. However, little is known either on the potential antibacterial mechanism between plant-derived compounds and organic acids.

Methyl salicylate which has the special flavor of holly leaves was an additive generally recognized as safe (GRAS) [22]. We speculated that the additive might have a similar effect as a flavoring agent or sweetener to improve the palatability and inductivity of feed or have an antioxidant capacity to help to maintain the quality of feed to promote FI. Consequently, the great growth performance in the CMT group was presented by alleviating the decrease of FI caused by weaning stress [23]. However, the COT group had no significant improvement on growth performance, which was consistent with an experiment of three diets with an oregano supplementation at 2 g, 4 g and 8 g per kg feed [24]. The beneficial effect of dietary phytobiotics could be influenced by its dosage and diet composition [25], so more tests are needed to verify the role of different dosage. In addition, there is a limitation that the solo encapsulation vehicle was not determined on the performance of piglets, which will be worth continuing investigation.

Serum biochemical indices can reflect growth performance, nutrition and health status to a certain extent. TP is composed of ALB and GLB. ALB revealed a significant correlation with body nutritional status, including ADG and feed conversion [26]. In this study, TP and ALB in the CMT group with better growth performance were significantly higher than the CON group. Consisted with the previous conclusion, in pigs, nutrition, body growth and ALB synthesis were interdependent and ALB was considered one of the most important predictors of performance, especially ADG [6]. The activities of GSH-PX and T-AOC increased significantly in the CMT group than in the CON group. The COT group had significantly higher T-AOC content than the CON group, which might result from the anti-oxidant capacity of the phenolic hydroxyl compounds in plant essential oils. The result was consistent with the previous report of inclusion of plant polyphenols to the diet [27]. T-AOC directly reflects the antioxidant capacity of the body, while enhanced SOD and GSH-PX would reduce the concentration of superoxide anion and peroxide in the body, as well as, lower concentration of serum MDA demonstrates the lower degree of oxidative damage took place in the body. Overall, diet supplemented with an encapsulated mixture of methyl salicylate and tributyrin could enhance the antioxidant capacity and accelerate the protein synthesis.

Weaning stress would damage gastrointestinal structure [28]. Villus length and crypt depth were correlated with the absorption surface and mucosal secretion [29]. Our results confirmed that the structure of ileum in both the CMT and COT groups had been improved and there was a positive relationship between serum antioxidant parameters and intestinal morphology, which was consistent with the previous study that intestinal injury was significantly reduced by SOD pretreatment [30]. The reason why the CMT group had better intestinal morphology might be attributed to phenolic hydroxyl groups, which have been reported to be beneficial to the maintenance of the intestinal integrity of piglets [31]. Another study has pointed out that tributyrin could strengthen the intestinal barrier via promoting cell differentiation [32]. A previous study showed that SCFAs could affect appetite and energy balance and regulate intestinal morphology [33]. SCFAs molecules could be absorbed by colonic epithelial cells and converted into energy to maintain the intestinal health of animals [34]. We examined the colonic SCFAs and found that valeric acid and isovaleric acid in the CMT group increased compared with the CON group, which was consistent with the conclusion of the previous literature data on the base diet supplemented with 1.5 g/kg organic acid and 30 mg/kg essential oils [35]. However, it was somewhat surprising since total SCFAs in the COT group appeared a tendency to decrease, which is worth investigating.

In terms of α-diversity, compared with the CON group, the Simpson index and Chao1 index showed opposite variation in the CMT group. The significance of this finding rests on the fact that there is still a great deal of controversy on the relationship between weight or obesity and intestinal microbial diversity [36]. However, the F/B ratio in the CMT group was increased, which was consistent with characteristics of obesity [37]. It is worth noting that the phyla *Proteobacteria* containing a wide variety of pathogens, including *Escherichia coli*, *Salmonella* and *Helicobacter pylori* had a relatively high abundance in the CON group. As reported by several studies, oregano essential oil could inhibit the activity of *Escherichia coli*s [38]. In line with this, the COT group showed a decrease in the abundance of *Proteobacteria* and *Escherichia/Shigella.* However, the antibacterial mechanism responsible for methyl salicylate in the animals’ intestine requires further discussion. Interestingly, compared with the CON group at the genus level, we found that the abundance of *Tissierella* decreased in both the COT and CMT groups, but the abundance of *Campylobacter* decreased only in the CMT group. Compared with positive bacteria, the structure of *Campylobacter* had stronger resistance to plant essential oils and was more sensitive to organic acids [39]. These different responses might allow for the discrepancy of the COT group and the CMT group on growth performance of piglets in this experiment. Moreover, as described by Qin et al. the drug resistance of *Campylobacter* in the intestinal tract of livestock and poultry in Asia was generally strong [40]. And through the correlation analysis, the increase of *Campylobacter* in the COT group might be the cause of the increase in the number of *Chlamydia*. Thus we tentatively linked the reason to the increasing abundance of *Chlamydia*, which were associated with different pathologies [41]. One hypothesis was that *Veillonellaceae* may be used as an index to measure the bactericidal ability of antimicrobials [42]. In this study, the lower abundance of *Veillonellaceae* indicated that the CMT group had a higher antimicrobial capacity. Additionally, a study found that microbial composition may not be a major factor determining differences in feed efficiency [43]. The CMT group had the higher FI but similar to feed to gain ratio compared with the CON group, which might cause by improving intestinal integrity [44].

Intestinal microorganisms could produce bioactive molecules which affect host metabolism [45]. PICRUST analysis showed that the genes related to fatty acid metabolism were significantly up-regulated in both the COT and CMT groups. Actually, butyric acid has been proved to improve the energy metabolism of mitochondrial cells through activating Adenosine 5‘-monophosphate (AMP)-activated protein kinase (AMPK), especially fatty acid oxidation [46]. These reports indicated that the supply of butyric acid in the form of tributyrin could alleviate the effect of weaning stress. Notably, in the CMT group, we also found that the quorum sensing of bacteria was interfered, which may reduce the drug resistance of bacteria by changing the ratio of Opp/Dpp about biofilm formation. Consistently, evidence confirmed that plant essential oils could destroy the quorum sensing of bacteria [47]. Whereas the information reflected by PICRUST was limited. Consequently, we applied GC-MS non-targeted metabolomics to explain the functional difference. Via the OPLS-DA model, we found that the metabolic level of the CMT group had significant difference compared with the CON group, while there was no significant difference in the metabolic level between the COT group and the CON group. The data showed that the concentrations of L-serine, L-aspartic acid and L-methionine in the CMT group changed significantly, which indicated bioavailability of amino acids and protein synthesis was improved. Pathway analysis showed that differential metabolites were enriched in cysteine and methionine metabolism, alanine, aspartic acid and glutamic acid metabolism, arginine biosynthesis and so on. Additionally, several reports pointed out that the synthesis of endogenous arginine related with growth performance and antioxidation of piglets [48,49].

Finally, we analyzed the correlation between differential bacteria and metabolites to reveal the interaction between microorganisms and hosts. Although there is too little information about *Streptophyta*, which was positively correlated with a variety of major metabolites. We also found that there was a high correlation between sorbitol and *Campylobacter*. The influence caused by a relative higher abundance of *Streptophyta* and *Campylobacter* on the host is worth being investigated more.

## 5. Conclusions

In conclusion, dietary inclusion of an encapsulated mixture of methyl salicylate and tributyrin improved the growth performance, antioxidant capacity, and intestinal villus morphology, and modulated the microbiota and their metabolites. The results may hopefully serve as useful information for the use of encapsulated mixtures of methyl salicylate and tributyrin as feed additives in piglets.

## Figures and Tables

**Table 1:** Basal diet ingredients and chemical composition.

Items, g/kg	
Ingredients	
Corn	597
Soybean meal	105
Fermented soybean meal	55
Extruded soybean	100
Fish meal	40
Whey powder	30
Glucose	20
Wheat flour	4.4
Soybean oil	10
L-Lysine hydrochloride	3
L-Threonine	1.5
DL-Methionine	0.6
Choline chloride	1
Sodium chloride	3.5
Calcium hydrophosphate	9
Limestone	10
Vitamin-mineral premix ^1^	10
Nutrition composition	
Digestible energy ^†^, MJ/kg (calculated)	143.96
Crude protein	190.05
Calcium	10.15
Lysine	13.37
Methionine	4.11

^1^ Provided per kilogram of diet: retinyl acetate, 1.2 MIU; cholecalciferol, 2700 IU; rac-α-tocopheryl acetate, 75 mg; menadione, 1.25 mg; thiamin, 1.5 mg; riboflavin, 2.5 mg; pantothenic acid, 40 mg; niacin, 34.4 mg; pyridoxol, 2.5 mg; biotin, 0.3 mg; folic acid, 3 mg; cobyrinic acid, 0.04 mg; Zn, 250 mg; Fe, 110 mg; Cu, 100 mg; Mn, 50 mg; I, 0.5 mg; Se, 0.5 mg. ^†^ Digestible energy was calculated.

## Supplementary Materials

The following are available online at https://www.mdpi.com/article/10.3390/microorganisms9061342/s1, Figure S1: Serum indices of different groups. (A) A/G. (B) LDH. (C) TC. (D) GLU. (E) ALT. (F) AST. (G) BUN. (H) UA. (I) SOD. Figure S2: The differences of gene expression among different groups analyzed by LEfSe. (A) LEfSe analysis based on Genomes (KEGG) ortholog (KO) database. (B) LEfSe analysis based on the MetaCyc database. (C) The expression of the top 20 KO genes respectively in different groups. Figure S3: Non-targeted metabonomics derived from the GC/MS metabolite profiles of ileum contents. (A) OPLS-DA score map of separate comparisons between the COT group and the CON group. (B) Significantly differential metabolites between the COT group and the CON group.

## Abbreviations

A/G: albumin/globulin; ALT, alanine aminotransferase; AST, aspartate aminotransferase; ALB, albumin; GLB, globulin; GLU, glucose; LDH, lactate dehydrogenase; TC, cholesterol; TG, triglyceride; TP, total protein; UA, uric acid; BUN, blood urea nitrogen; MDA, methylene dioxyamphetamine; SOD, superoxide dismutase; GSH-PX, glutathione peroxidase; T-AOC, blood total antioxidant capacity; VH/CD, villus height/crypt depth; SCFAs, short-chain fatty acids; ADG, average daily gain; FI, feed intake; OTUs, operational taxonomic units; F/B, *Firmicutes*/*Bacteroidetes*; g, genus.
