# Supplementary figures and images for: Encapsulated Mixture of Methyl Salicylate and Tributyrin Modulates Intestinal Microbiota and Improves Growth Performance of Weaned Piglets
Source: Microorganisms. 2021 Jun 21;9(6):1342. doi: 10.3390/microorganisms9061342 (PMC8235159; doi:10.3390/microorganisms9061342)

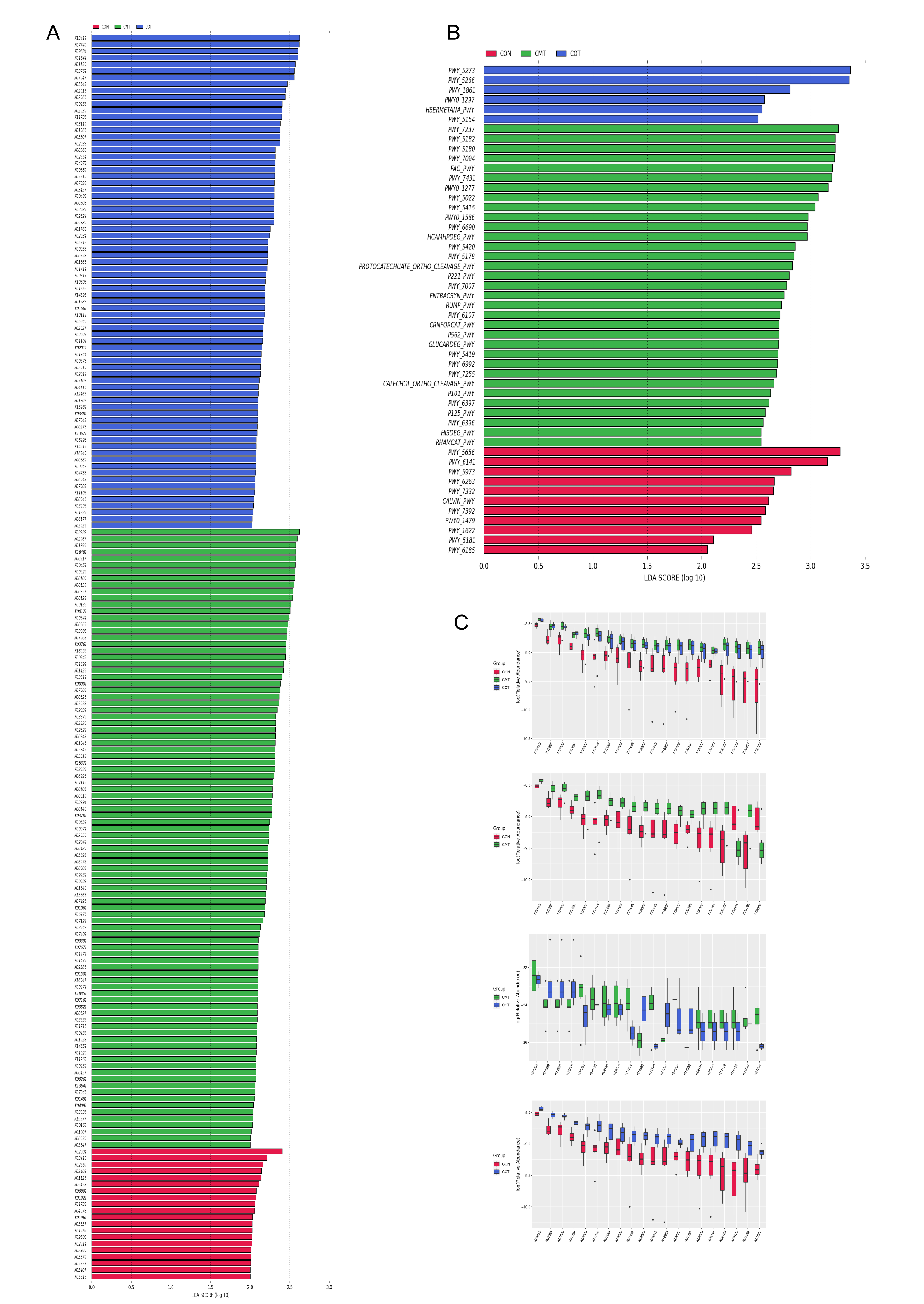

Supplement: Supplementary file 1 [file microorganisms-09-01342-s001.zip › FigureS2.tif]

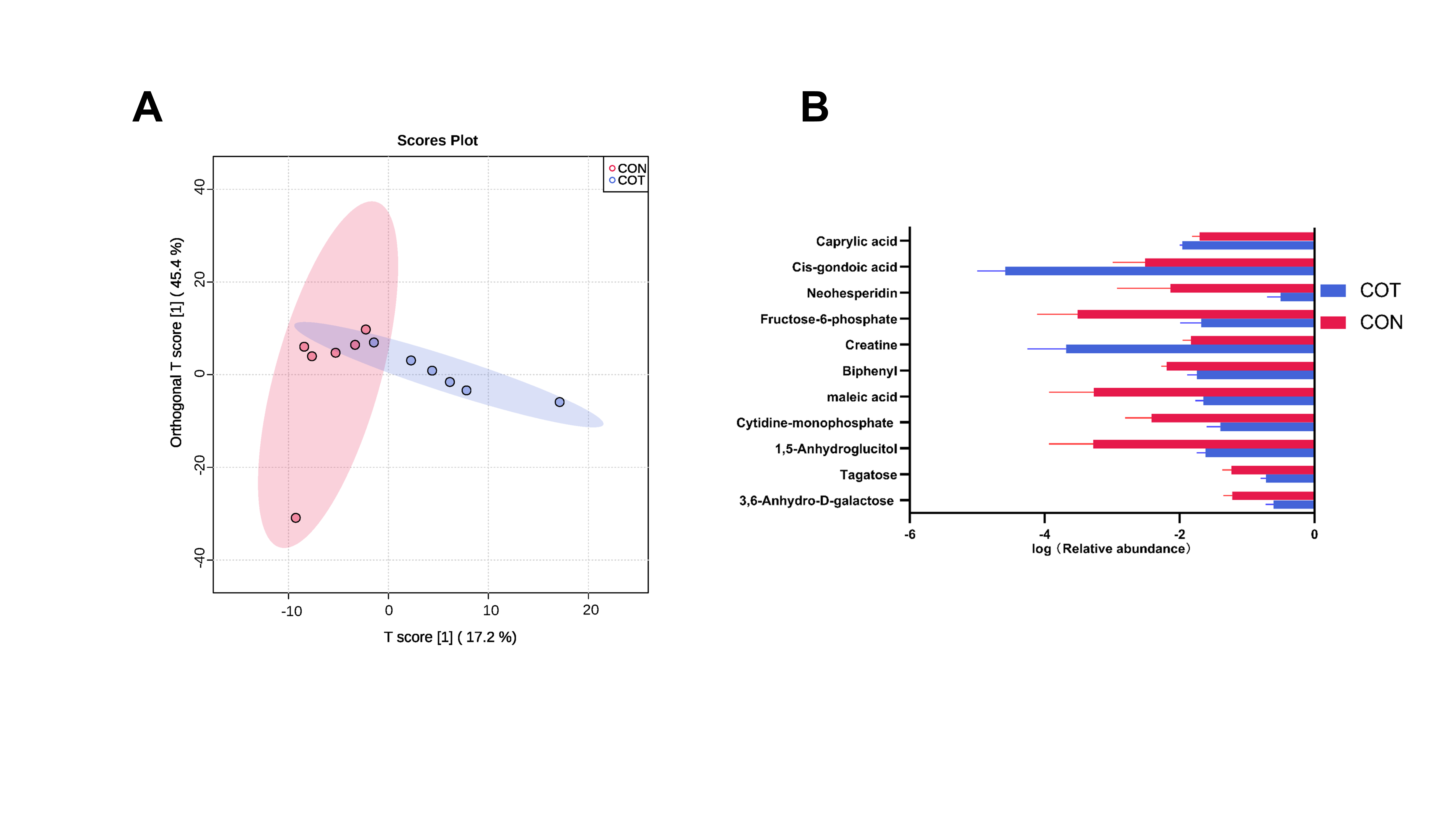

Supplement: Supplementary file 1 [file microorganisms-09-01342-s001.zip › FigureS3.tif]

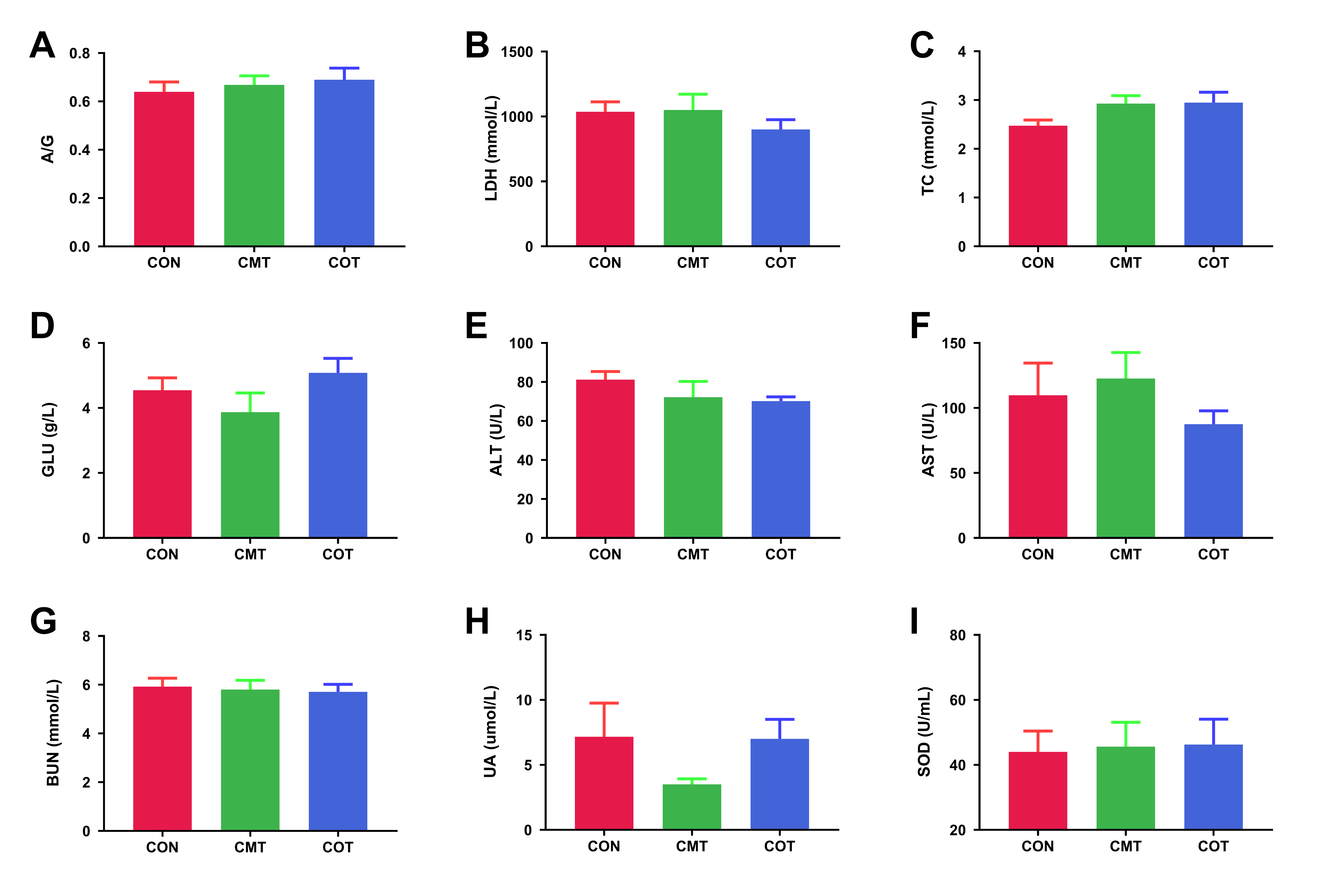

Supplement: Supplementary file 1 [file microorganisms-09-01342-s001.zip › FigureS1.tif]
